# Risk Calculators in Bipolar Disorder: A Systematic Review

**DOI:** 10.3390/brainsci10080525

**Published:** 2020-08-06

**Authors:** Joana Silva Ribeiro, Daniela Pereira, Estela Salagre, Manuel Coroa, Pedro Santos Oliveira, Vítor Santos, Nuno Madeira, Iria Grande, Eduard Vieta

**Affiliations:** 1 Psychiatry Department, Centro Hospitalar Vila Nova de Gaia/Espinho, 4434-502 Vila Nova de Gaia, Portugal; 2 Faculty of Medicine, Institute of Psychological Medicine, University of Coimbra, 3004-504 Coimbra, Portugal; dsmpereira4@gmail.com (D.P.); coroaofc@gmail.com (M.C.); pedrosantosoliveira89@gmail.com (P.S.O.); vitorsantos74@gmail.com (V.S.); nunogmadeira@gmail.com (N.M.); 3 Psychiatry Department, Centro Hospitalar e Universitário de Coimbra, 3000-075 Coimbra, Portugal; 4 Bipolar and Depressive Disorders Unit, Institute of Neurosciences, Hospital Clinic, University of Barcelona, IDIBAPS, CIBERSAM, Barcelona, 08035 Catalonia, Spain; ESALAGRE@clinic.cat (E.S.); EVIETA@clinic.cat (E.V.); 5 Coimbra Institute for Biomedical Imaging and Translational Research, University of Coimbra, 3000-548 Coimbra, Portugal

**Keywords:** bipolar disorder, bipolar depression, risk prediction, risk calculator, risk score

## Abstract

Introduction: Early recognition of bipolar disorder improves the prognosis and decreases the burden of the disease. However, there is a significant delay in diagnosis. Multiple risk factors for bipolar disorder have been identified and a population at high-risk for the disorder has been more precisely defined. These advances have allowed the development of risk calculators to predict individual risk of conversion to bipolar disorder. This review aims to identify the risk calculators for bipolar disorder and assess their clinical applicability. Methods: A systematic review of original studies on the development of risk calculators in bipolar disorder was performed. The studies’ quality was evaluated with the Newcastle-Ottawa Quality Assessment Form for Cohort Studies and according to recommendations of the Transparent Reporting of a multivariable prediction model for Individual Prognosis or Diagnosis Initiative. Results: Three studies met the inclusion criteria; one developed a risk calculator of conversion from major depressive episode to bipolar disorder; one of conversion to new-onset bipolar spectrum disorders in offspring of parents with bipolar disorder; and the last one of conversion in youths with bipolar disorder not-otherwise-specified. Conclusions: The calculators reviewed in this article present good discrimination power for bipolar disorder, although future replication and validation of the models is needed.

## 1. Introduction

Bipolar disorder (BP) is a common, chronic, and highly morbid illness characterized by hypomanic/manic and depressive episodes, which often runs a relapsing and remitting course, affecting 2–3% of the general population worldwide [1,2]. Usually, BP onset occurs during adolescence or early adult years (mean age ~20 years old), that is, before or during the most productive period of adulthood [3,4].

Although it is largely recognized that an early intervention improves the prognosis and decreases the burden of the disease, there is still an important delay between illness onset and diagnosis, with an average delay of 5–10 years [5,6]. One of the major diagnostic difficulties is to differentiate BP from unipolar depression. This difficulty is due to several factors, such as: (1) first mood episode is depressive in half of the patients; (2) bipolar patients spend more time with depressive symptoms than with manic symptoms; (3) the search for help is more frequent in depressive episodes; (4) hypomanic episodes or mixed symptoms often go unrecognized [5,6,7,8,9]. Sometimes patients with bipolar disorder may also be misdiagnosed with other psychiatric disorders, such as psychotic or substance use disorders, whenever psychotic symptoms or substance misuse prevail and mask affective symptoms [10,11]. Additionally, patients with an onset of disease before 18 years old are more likely to have longer delays in diagnosis and first intervention, due to a greater difficulty for the clinicians to identify less severe mood variations during childhood and adolescence, as well as the greater frequency of a depressive onset in those patients [2,3,5]. As both longer periods of untreated illness and an early onset have been related with poorer prognosis of the disease, the diagnostic delay in this subgroup of patients is of particular importance [3,4,12].

Prevention and early intervention are major goals of modern medicine. In this context, clinical staging models for mental illness, including BP, have been created. These staging models, based on the hypothesis of neuroprogression in mental disorders, establish a basis for therapeutic intervention strategies in the different illness stages, including the early ones [13]. Evidence suggests the existence of a period of subthreshold and nonspecific symptoms (prodrome) before the full manifestation of the illness [14]. The most replicated finding to date has been the presence of subthreshold manic symptoms prior to the first full-blown manic episode [15]. A recent meta-analysis, of early manifestations of BP in youth, however, found a variety of prodromal symptoms in this population, the most frequent being increased energy, diminished ability to think, indecision, pressured speech, talkativeness, elated mood, academic or work difficulties, insomnia, depressed mood, and increased goal-directed activities [16]. Hence, they warn that the prodromal period appears to be heterogeneous and thus highlight the need of an individualized approach when assessing prodromes of bipolar disorder. Different clinical and sociodemographic factors also have been pointed out as predictors of a higher risk of progression to BP in depressed patients, the most consistent being a family history of BP, earlier age at onset, presence of psychotic symptoms, atypical depressive symptoms, and subthreshold manic symptoms [7,17].

Although many risk factors for conversion to BP have been identified, there is a need to develop tools that are easy to access and use, such as risk calculators, that allow clinicians to quantify the individual risk of conversion to BP and support them in choosing more specific therapeutic approaches [13]. 

Multiple models of risk calculation have been developed in different medical areas, such as cardiovascular diseases and cancer, which allowed the identification of risk populations and the implementation of screening programs and early intervention measures [2]. Risk calculators are clinical instruments developed based on the data available for a particular disease, identifying the ideal set of clinical factors that makes it possible to estimate the likelihood that an individual will develop a specific condition in the future. [18] They make it possible to derive the risk forecast for an individual, using a multivariate model based on the disease’s progression in a large sample of patients. Through imputation, calculators can accommodate incomplete information about risk indicators, complementing the traditional clinical assessment. Nonetheless, they become more reliable, with a narrower range of certainty and the more complete the information available in a given case. [19] Currently, predictive models must accurately reflect existing patterns in the underlying data, being valid when the data are comparable and replicable in different samples. Several factors can contribute to a low predictive robustness and replicability of a model, such as a high frequency of missing data or a small set of data in the sample used for the development of the model [20].

Despite the multiple existing studies on risk factors, calculation models in psychiatry are still scarce, the most commonly studied being those evaluating the risk for developing psychosis or schizophrenia in prodromal samples [21,22,23]. 

This study aims to review all the risk calculators developed for BP, namely, what variables they evaluated, their predictive value, and their main limitations. We sought as well to provide a critical analysis of the current state of knowledge in the area, as well as to establish starting points for the elaboration of other models with applicability in clinical practice.

## 2. Materials and Methods

We performed a systematic review according to Preferred Reporting Items for Systematic Review and Meta-Analysis (PRISMA) guidelines [24]. 

Original studies on risk calculators for conversion to BP in at-risk populations, using a set of clinical variables and/or biomarkers available on clinical practice, written in English, Portuguese or Spanish languages, and published in a scholarly peer-reviewed journal were eligible for this review, with no year or country restriction. We identified the studies by searching relevant papers via *PubMed/MEDLINE* (http://www.ncbi.nlm.nih.gov/pubmed) and *Embase* (https://www.embase.com) using the following keywords: (“bipolar disorder” OR “mania” OR “bipolar depression”) AND (“prediction” OR “risk prediction” OR “prediction models” OR “predictive model” OR “risk score” OR “risk calculator”). The last article search was on April 2020.

Each study’s title and abstract were screened for eligibility by the first and second authors; subsequently, full texts of all potentially relevant studies were revised and examined for eligibility. We analyzed the included studies and extracted information about: (a) country in which data were collected, (b) participants’ characteristics, (c) number of subjects included, (d) follow-up time, (e) type of variables included in the risk calculator, (f) main results, (g) main conclusions, (h) limitations, and (i) risk of bias. 

The quality of the studies selected for review was evaluated with the *Newcastle-Ottawa Quality Assessment Form for Cohort Studies* that assesses selection, comparability and outcome/exposure domains [25].

The quality of the risk models’ development and validation was assessed in accordance with the recommendations of the *Transparent Reporting of a multivariable prediction model for Individual Prognosis Or Diagnosis (TRIPOD) Initiative* [26].

## 3. Results

**Figure 1:**
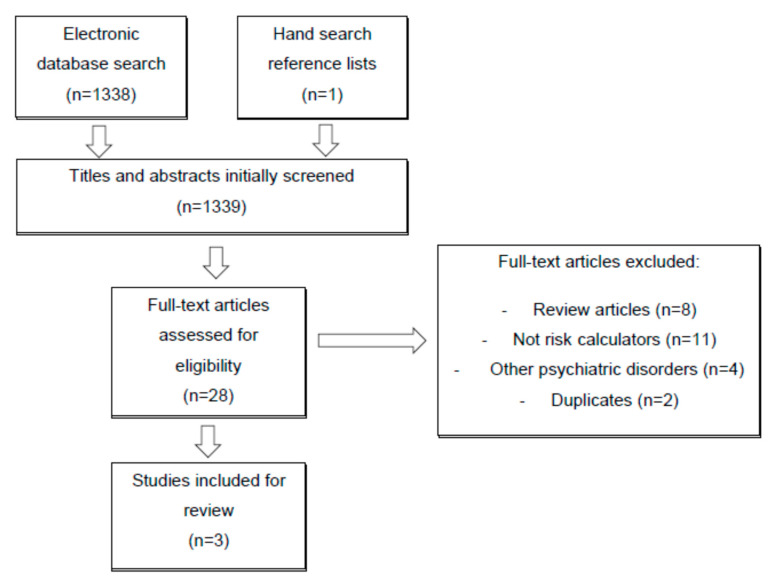
Preferred Reporting Items for Systematic Review and Meta-Analysis (PRISMA) flow diagram of the study selection process

A total of 1339 articles were initially screened by title and abstract, with 28 selected for full-text reading. After the full-text review, only three studies met the eligibility criteria for inclusion in this systematic review. One study was from China [27] and the other two were from the United States of America [18,28]. In Figure 1, the PRISMA flow diagram is presented, providing more detailed information regarding the selection process.

Gan et al. developed a risk calculator for conversion from major depressive episode to BP from a sample of patients diagnosed with a depressive episode and followed for one year in an outpatient clinic [27]. This calculator uses six clinical variables: age of onset, maximum duration of depressive episodes, somatalgia, hypersomnia, diurnal variation of mood, and irritability. In a one-year follow-up of 344 patients diagnosed with depressive episode, those variables were the ones with higher predictive value and therefore included in their instrument, with an AUC of 0.85, a sensitivity of 75%, and a specificity of 83%.

The study of Hafeman et al. included offspring of patients with BP I or II recruited from The Pittsburgh Bipolar Offspring Study and elaborated a risk calculator for assessing the probability of developing new-onset bipolar spectrum disorders (BPSD) [28]. Their model uses seven clinical variables: mania, depression, anxiety, emotional lability, functioning, age at visit, and parental age of BP onset. Four different risk score cutoffs were established and the positive predictive value, sensitivity, and specificity for each one were presented (as shown in Table 1). 

In another study from the same group, Birmaher et al. recruited youths with BP Not-Otherwise-Specified (BP-NOS) from the Course and Outcome of Bipolar Youth (COBY) study and developed a risk calculator of conversion to BP-I or II [18]. This model is based on ten demographic and clinical variables (mania, depression, anxiety, emotional lability, functioning, duration of illness, age, race, gender, and family history), with an AUC of 0.71. The study was externally validated in a sample from The Pittsburgh Bipolar Offspring Study, with an even stronger discrimination (AUC = 0.75).

All data regarding the variables included in each calculator, their predictive value, and limitations are shown in Table 1.

Table 2 shows the studies’ quality assessment based on the Newcastle-Ottawa Quality Assessment Form for Cohort Studies. This instrument assesses the quality of non-randomized studies with a star system evaluating three perspectives: (1) selection of the study groups, (2) comparability of the groups, and (3) the outcome of interest. All studies were evaluated as being of good quality, although all three present a risk of significant selection bias, since the sample is obtained from selected groups.

Table 3 presents the quality assessment according with TRIPOD initiative recommendations. According to these recommendations, generally all models have good reporting quality, although none of them explains clearly how to use the risk calculator. The study by Birmaher et al. is the only one which was externally validated [18].

## 4. Discussion

Risk prediction models are useful tools to guide the clinicians in decision making, regarding the risk to develop a certain medical condition and its individual management [29]. Risk calculators estimate the probability of an individual to develop a particular outcome based on different predictors, such as demographic variables, clinical evaluation, and complementary diagnostic exam results [30]. In the last decades, risk prediction models have been proposed in different areas of medical knowledge. The Framingham Study on cardiovascular disorders is, probably, the best-known example of risk prediction models in medicine, predicting the cardiovascular risk [28,29]. 

In psychiatry, the development of risk prediction models becomes more challenging, due to the absence of easily quantifiable diagnostic parameters, but, at the same time, its potential value is even higher than in other areas of medicine. Precision psychiatry should integrate different sources of information about the individual, such as biographical, clinical, and biological data [31]. The fact that there is still much to understand about the etiopathological mechanisms and the lack of reliable biomarkers for psychiatric disorders contribute to the paucity of clinical risk prediction models in mental illness [32]. Consequently, psychiatry has traditionally focused more on the development of treatments that minimize the consequences of the disease than on prevention and early intervention [33]. 

Most studies of risk factors for bipolar disorder focus on examining the risk in an entire group rather than quantifying an individual’s risk of having that disorder, which is essential to advance through personalized monitoring and treatment strategies [22]. In that regard, analyzing risk prediction models and building risk calculators are essential initial steps toward advancing individualized treatment and eventually, targeted prevention strategies to reduce an individual’s risk [34].

Several studies have identified multiple risk factors for the development of BP, such as family history or atypical depressive symptoms [33]. In addition, the growing knowledge about the pathogenesis and pathophysiology of the disease over the past few years has allowed the identification of potential biomarkers that may become important assistants in the differential diagnosis [6].

Some biomarkers have been found to be differentially altered in BP patients and healthy controls, like high-sensitivity C-reactive protein, interleukin-6, brain derived neurotrophic factor or tumor necrosis factor (TNF)-α, and, more recently, serum uric acid levels, have proven useful as a predictor of bipolarity in individuals with a major depressive episode [31,35,36,37]. 

Despite the increasing knowledge about risk factors and biomarkers in BP, findings are sometimes contradictory, which limits their usefulness in clinical practice. Therefore, it is important to systematize information and create accessible tools, easy to use, on daily basis, in a clinical setting. 

In this study, we reviewed the existing risk calculators of conversion for BP. As shown in the results section, although there are numerous studies that point out various risk factors for the development of bipolar disorder, only three risk calculators were found. Therefore, these results show the lack of risk quantification models in mental illness.

Despite recent advances in the field of genetics, peripheral, and neuroimaging markers, all three studies reviewed have calculators based on sociodemographic and clinical variables [31,34,35,36,37,38]. Despite this, all the risk calculators presented predictive values that are quite promising and comparable to those of risk calculators in other areas of medicine, such as cardiovascular diseases [18,27,28,29].

Although it lacks replication and external validation, the study by Gan et al. shows good results, with an AUC of 0.85, a sensitivity of 75%, and a specificity of 83%. In addition, the lack of information regarding the questionnaire used to assess the variables, which was developed by the researchers, is a major limitation [27]. 

The studies of Hafeman et al. and Birmaher et al. have the advantage of establishing different risk score cutoffs, presenting the positive predictive value, sensitivity, and specificity for each one, which can be useful in stratifying risk at different levels and the consequent adaptation of early intervention strategies for each at-risk individual. However, these two calculators have been developed in BP at-risk populations, and it is unknown how they would perform in youth without a family history or with BP-NOS. The study by Birmaher et al. was the only one that was externally validated in a sample from The Pittsburgh Bipolar Offspring Study, with an even stronger discrimination than the original population. 

Despite their good results, the risk calculators reviewed here still need to be replicated and externally validated in different populations, since they were all developed in selected populations and are potentially not representative of the population that we usually deal with in clinical practice, due to the risk of selection bias [35,39]. In fact, although calculators give the clinician an estimate of individuals with a higher or lower risk of developing BP, their implementation should always be complemented with a detailed clinical assessment. The risk calculators may be useful as a screening in populations considered at risk for the development of BP, allowing the identification of individuals who need closer monitoring in order to reduce the diagnostic delay and allow an early intervention. However, these tools cannot be used in isolation, since the individual pattern of symptoms, as well as their temporal evolution, are essential for proper and truly personalized diagnosis and intervention [31].

One limitation of our study was the exclusion of articles published in languages other than English, Portuguese, and Spanish. Moreover, due to the scarce research on this topic and the heterogeneity in study design, we were not able to conduct a meta-analysis that would have been useful to provide important information regarding the predictive power of the existing models.

## 5. Conclusions

In the future, it is possible that new risk calculators will include not only sociodemographic and clinical variables, but also some biomarkers, which may contribute to an even greater predictive value. Future research should also focus on the replication and validation of risk prediction models, and in making them useful and easily applicable in clinical practice.

## Figures and Tables

**Table 1:** Summary of the articles on developing risk prediction models for bipolar disorder (BP).

Article	Sample Characteristics	Variables Included in the Model	Variables Assessment	Risk Prediction Statistics	Classification Statistics	Main Conclusions/Outcomes	Limitations
Gan et al. (2011)	N = 344 patients with major depressive episodes; 268 completing 1-year follow-up Proportion of the outcome = 63% (n = 169)	Age at first onset Maximum duration of depressive episodes Somatalgia Hypersomnia Diurnal variation of mood Irritability	Questionnaire designed by the researchers	SRD = 0.70 AUC = 0.85	PPV = 0.87 NPV = 0.67 SEN = 75% SPE = 83%	The model based on six clinical characteristics robustly predict the transition from major depressive episodes to BP.	Short follow-up period; possibility of selection bias; the anti-depressant treatment was not assessed; without external validation.
Hafeman et al. (2017)	N = 412 Bipolar at-risk (offspring of BP I and II) completing 5-year follow-up	Mania Depression Anxiety Emotional lability Functioning Offspring age at visit Parental age at mood disorder onset	1. Modified K-SADS Mania Rating Scale (KMRS) 2. Depression items from K-SADS–Present Version (KDRS)-Modified 3. SCARED-Screen for Child Anxiety Related Emotional Disorders (child reported) 4. CALS-Children’s Affective Lability Scale (child reported) 5. CGAS-Children’s Global Assessment Scale	AUC = 0.76	Risk Score Cutoff 0.5: PPV = 0.15 SEN = 0.82 SPE = 0.49 Risk Score Cutoff 0.10: PPV = 0.22 SEN = 0.53 SPE = 0.80 Risk Score Cutoff 0.15: PPV = 0.30 SEN = 0.37 SPE = 0.91 Risk Score Cutoff 0.20: PPV = 0.32 SEN = 0.21 SPE = 0.95	A model based on anxiety, manic symptoms, depressive symptoms, mood lability, poor general psychosocial functioning, and earlier parental age at onset individually and collectively assessed the probability of new-onset BPSD within the next 5 years in a population at familial risk for BP.	Few youths were diagnosed with BP I or II; Follow-up visits scheduled every 2 years without external validation.
Birmaher et al. (2018)	N = 140 BP-NOS; 120 completing 5-year follow-up	Mania Depression Anxiety Emotional lability Functioning Duration of Bipolar Illness (Years) Child’s Age Race Family History of Mania Gender	1. Modified KMRS (K-SADS Mania Rating Scale) 2. Modified KDRS (K-SADS–Present Version) 3. SCARED-Screen for Child Anxiety Related Emotional Disorders (child reported) 4. CALS-Children’s Affective Lability Scale (child reported) 5. CGAS-Children’s Global Assessment Scale	AUC = 0.71	Risk Score Cutoff 0.20: PPV = 0.46 NPV = 0.85 SEN = 0.86 SPE = 0.44 Risk Score Cutoff 0.25: PPV = 0.52 NPV = 0.81 SEN = 0.75 SPE = 0.61 Risk Score Cutoff 0.30: PPV = 0.56 NPV = 0.77 SEN = 0.62 SPE = 0.72 Risk Score Cutoff 0.35: PPV = 0.60 NPV = 0.73 SEN = 0.47 SPE = 0.82 Risk Score Cutoff 0.40: PPV = 0.65 NPV = 0.71 SEN = 0.36 SPE = 0.89	A model based on family history of hypo/mania and elevated levels of manic, mood lability, and anxiety symptoms can predict the conversion risk from BP-NOS to BP I or II in patients ages 6–17 years old. Results were externally validated in a sample recruited from the community (BIOS) with an even stronger discrimination (75%). If the conversion did not occur within four years of the initial BP-NOS diagnosis, the risk dropped considerably.	Majority of participants were Caucasian recruited from clinical settings; the presence of factors associated with high-risk for conversion are not stable and may change over time

Abbreviations: AUC = area under the curve; BIOS = Pittsburgh Bipolar Offspring Study; BP: Bipolar Disorder; BPSD: bipolar spectrum disorder; BP-NOS: Bipolar Disorder Not-Otherwise-Specified; NPV = negative predictive value; PPV = positive predictive value; SEN = sensitivity; SPE = specificity; SRD = success rate difference.

**Table 2:** Quality Assessment based on Newcastle-Ottawa Quality Assessment Form for Cohort Studies.

		Gan et al.	Hafeman et al.	Birmaher et al.
**SELECTION**	1. Representativeness of the exposed cohort	Selected group	Selected group	Selected group
	2. Selection of the non-exposed cohort	Same community as the exposed cohort (⁕)	Same community as the exposed cohort (⁕)	Same community as the exposed cohort (⁕)
	3. Ascertainment of exposure	Structured interview (⁕)	Structured interview (⁕)	Structured interview (⁕)
	4. Demonstration that outcome of interest was not present at start of study	Yes (⁕)	Yes (⁕)	Yes (⁕)
**COMPARABILITY**	1. Comparability of cohorts based on design or analysis controlled for confounders	Study controls for age and other different sociodemographic and clinical factors (⁕⁕)	Study controls for other different sociodemographic and clinical factors (⁕)	Study controls for other different sociodemographic and clinical factors (⁕)
**OUTCOME**	1. Assessment of outcome	Independent blind assessment (⁕)	No description	Independent blind assessment (⁕)
	2. Follow-up long enough for outcomes to occur (Indicate the median duration)	No (1 year follow-up)	Yes (⁕) (median of 9.5 years)	Yes (⁕) (median of 11.5 years)
	3. Adequacy of follow-up of cohorts	Subjects lost to follow up unlikely to introduce bias (⁕)	No statement	No statement
	**RESULT**	**Good Quality**	**Good Quality**	**Good Quality**

**Table 3:** Quality assessment of the calculators according to the Transparent Reporting of a multivariable prediction model for Individual Prognosis Or Diagnosis (TRIPOD) checklist.

Section/Topic	Item	Checklist Item	Gan et al.	Hafeman et al.	Birmaher et al.
**Title and abstract**					
Title	1	Identify the study as developing and/or validating a multivariable prediction model, the target population, and the outcome to be predicted.	✓	✓	✓
Abstract	2	Provide a summary of objectives, study design, setting, participants, sample size, predictors, outcome, statistical analysis, results, and conclusions.	✓	✓	✓
**Introduction**					
Background and objectives	3a	Explain the medical context (including whether diagnostic or prognostic) and rationale for developing or validating the multivariable prediction model, including references to existing models.	✓	✓	✓
	3b	Specify the objectives, including whether the study describes the development or validation of the model or both.	✓	✓	✓
**Methods**					
Source of data	4a	Describe the study design or source of data (e.g., randomized trial, cohort, or registry data), separately for the development and validation data sets, if applicable.	✓	✓	✓
	4b	Specify the key study dates, including start of accrual; end of accrual; and, if applicable, end of follow-up.	✓	✓	✓
Participants	5a	Specify key elements of the study setting (e.g., primary care, secondary care, general population) including number and location of centers.	✓	✓	✓
	5b	Describe eligibility criteria for participants.	✓	✓	✓
	5c	Give details of treatments received, if relevant.	n/a	n/a	n/a
Outcome	6a	Clearly define the outcome that is predicted by the prediction model, including how and when assessed.	✓	✓	✓
	6b	Report any actions to blind assessment of the outcome to be predicted.	✓	✓	✓
Predictors	7a	Clearly define all predictors used in developing or validating the multivariable prediction model, including how and when they were measured.	✓	✓	✓
	7b	Report any actions to blind assessment of predictors for the outcome and other predictors.	✓	✓	✓
Sample size	8	Explain how the study size was arrived at.	✓	✓	✓
Missing data	9	Describe how missing data were handled (e.g., complete-case analysis, single imputation, multiple imputation) with details of any imputation method.	✓	✓	✓
Statistical analysis methods	10a	Describe how predictors were handled in the analyses	✓	✓	✓
	10b	Specify type of model, all model-building procedures (including any predictor selection), and method for internal validation	✓	✓	✓
	10c	For validation, describe how the predictions were calculated.	n/a	n/a	✓
	10d	Specify all measures used to assess model performance and, if relevant, to compare multiple models.	✓	✓	✓
	10e	Describe any model updating (e.g., recalibration) arising from the validation, if done.	n/a	n/a	n/a
Risk groups	11	Provide details on how risk groups were created, if done.	n/a	✓	✓
Development vs. validation	12	For validation, identify any differences from the development data in setting, eligibility criteria, outcome, and predictors.	n/a	n/a	✓
**Results**					
Participants	13a	Describe the flow of participants through the study, including the number of participants with and without the outcome and, if applicable, a summary of the follow-up time. A diagram may be helpful.	✓	✓	✓
	13b	Describe the characteristics of the participants (basic demographics, clinical features, available predictors), including the number of participants with missing data for predictors and outcome.	✓	✓	✓
	13c	For validation, show a comparison with the development data of the distribution of important variables (demographics, predictors and outcome).	n/a	n/a	✓
Model development	14a	Specify the number of participants and outcome events in each analysis	✓	✓	✓
	14b	If done, report the unadjusted association between each candidate predictor and outcome	✓	✓	✓
Model specification	15a	Present the full prediction model to allow predictions for individuals (i.e., all regression coefficients, and model intercept or baseline survival at a given time point)	✓	✓	✓
	15b	Explain how to use the prediction model	✓	✓	✓
Model performance	16	Report performance measures (with CIs) for the prediction model	✓	✓	✓
Model updating	17	If done, report the results from any model updating (i.e., model specification, model performance)	n/a	n/a	n/a
**Discussion**					
Limitations	18	Discuss any limitations of the study (such as nonrepresentative sample, few events per predictor, missing data).	✓	✓	✓
Interpretation	19a	For validation, discuss the results with reference to performance in the development data, and any other validation data.	n/a	n/a	✓
	19b	Give an overall interpretation of the results, considering objectives, limitations, results from similar studies, and other relevant evidence.	✓	✓	✓
Implications	20	Discuss the potential clinical use of the model and implications for future research.	✓	✓	✓
**Other information**					
Supplementary information	21	Provide information about the availability of supplementary resources, such as study protocol, Web calculator, and data sets.	✓	✓	✓
Funding	22	Give the source of funding and the role of the funders for the present study.	✓	✓	✓

## References

[1] Mantin D.J., Smithh D.J. (2013). Is there a clinical prodrome of bipolar disorder? A review of the evidence. Expert Rev..

[2] Bauer M., Andreassen O.A., Geddes J.R., Kessing L.V., Lewitzka U., Schulze T.G., Vieta E. (2018). Areas of uncertainties and unmet needs in bipolar disorders: Clinical and research perspectives. Lancet Psychiatry.

[3] Vieta E., Berk M., Schulze T.G. (2018). Bipolar disorders. Nat. Rev. Dis Prim..

[4] Hartmann J.A., Nelson B., Ratheesh A., Treen D., Mcgorry P.D. (2018). At-risk studies and clinical antecedents of psychosis, bipolar disorder and depression: A scoping review in the context of clinical staging. Psychol. Med..

[5] Dagani J., Signorini G., Nielssen O., Bani M., Pastore A., De Girolamo G., Large M. (2017). Meta-analysis of the Interval between the onset and management of bipolar disorder. Can. J. Psychiatry.

[6] Grande I., Berk M., Birmaher B., Vieta E. (2016). Bipolar Disorder. Lancet.

[7] Phillips M.L., Kupfer D.J. (2013). Bipolar disorder diagnosis: Challenges and future directions. Lancet.

[8] Malhi G.S., Bargh D.M., Coulston C.M., Das P., Berk M. (2014). Predicting bipolar disorder on the basis of phenomenology: Implications for prevention and early intervention. Bipolar Disord..

[9] Sup Y., Hee I., Wang H., Rim H. (2015). A diagnosis of bipolar spectrum disorder predicts diagnostic conversion from unipolar depression to bipolar disorder: A 5-year retrospective study. J. Affect. Disord..

[10] Altamura A.C., Buoli M., Caldiroli A., Caron L., Melter C.C., Dobrea C., Cigliobianco M., Quarantini F.Z. (2015). Misdiagnosis, duration of untreated illness (DUI) and outcome in bipolar patients with psychotic symptoms: A naturalistic study. J. Affect. Disord..

[11] Singh T., Rajput M. (2006). Misdiagnosis of bipolar disorder. Psychiatry.

[12] Murru A., Primavera D., Oliva M., Meloni M.L., Vieta E., Carpiniello B. (2015). The role of comorbidities in duration of untreated illness for bipolar spectrum disorders. J. Affect. Disord..

[13] Pinna M., Manchia M. (2014). Prognostic models in bipolar disorder: Can the prediction of the long-term clinical course rely on the integration of clinical and molecular data?. Biomark. Med..

[14] Faedda G.L., Baldessarini R.J., Marangoni C., Bechdolf A., Berk M., Birmaher B., Conus P., DelBello M.P., Duffy A., Hillegers M.H.J. (2019). An international society of bipolar disorders task force report: Precursors and prodromes of bipolar disorder. Bipolar Disord..

[15] Vieta E., Salagre E., Grande I., Carvalho A.F., Fernandes B.S., Berk M., Birmaher B., Tohen M., Suppes T. (2018). Early intervention in bipolar disorder. Am. J. Psychiatry.

[16] Van Meter A.R., Burke C., Youngstrom E.A., Faedda G.L., Christoph U., Correll M. (2016). The bipolar prodrome meta-analyses of symptom prevalence prior to initial or recurrent mood episodes. J. Am. Acad Child. Adolesc. Psychiatry.

[17] Ratheesh A., Davey C., Hetrick S., Alvarez-Jimenez M., Voutier C., Bechdolf A., McGorry P.D., Scott J., Berk M., Cotton S.M. (2017). A systematic review and meta-analysis of prospective transition from major depression to bipolar disorder. Acta Psychiatr. Scand..

[18] Birmaher B., Merranko J.A., Goldstein T.R., Gill M.K., Goldstein B.I., Hower H., Yen S., Hafeman D., Strober M., Diler R.S. (2018). A risk calculator to predict the individual risk of conversion from subthreshold bipolar symptoms to bipolar disorder I or II in youth. J. Am. Acad Child. Adolesc. Psychiatry.

[19] Cannon T.D., Yu C., Addington J., Bearden C.E., Cadenhead K.S., Cornblatt B.A., Heinssen R., Jeffries C.D., Mathalon D.H., McGlashan T.H. (2016). An individualized risk calculator for research in prodromal psychosis. Am. J. Psychiatry.

[20] Harrell F.E., Lee K.L., Mark D.B. (1996). Multivariable prognostic models: Issues in developing models, evaluating assumptions and adequacy, and measuring and reducing errors. Stat. Med..

[21] Vigo D., Thornicroft G., Atun R. (2016). Estimating the true global burden of mental illness. Lancet Psychiatry.

[22] Bernardini F., Attademo L., Cleary S.D., Luther C., Shim R.S., Quartesan R., Compton M.T. (2017). Risk prediction models in psychiatry: Toward a new frontier for the prevention of mental illness. J. Clin. Psychiatry.

[23] Fusar-Poli P., Werbeloff N., Rutigliano G., Oliver D., Davies C., Stahl D., McGuire P., Osborn D. (2019). Transdiagnostic risk calculator for the automatic detection of individuals at risk and the prediction of Psychosis: Second replication in an independent national health service trust. Schizophr Bull..

[24] Moher D., Liberati A., Tetzlaff J., Altman D.G., Group T.P. (2009). Preferred reporting items for systematic reviews and meta-analyses: The PRISMA statement. PLoS Med..

[25] Wells G.A., Shea B., O’Connell D., Robertson J., Peterson J., Welch V., Losos M., Tugwell P. (2013). The Newcastle-Ottawa Scale (NOS) for Assessing the Quality of Nonrandomized Studies in Meta-Analyses.

[26] Macaskill P., Steyerberg E.W., Vickers A.J., Ransohoff D.F., Collins G.S. (2015). Transparent reporting of a multivariable prediction model for individual prognosis or diagnosis ( TRIPOD ): Explanation and elaboration. Ann. Intern. Med..

[27] Gan Z., Diao F., Wei Q., Wu X., Cheng M., Guan N., Zhang M., Zhang J.-B. (2011). A predictive model for diagnosing bipolar disorder based on the clinical characteristics of major depressive episodes in Chinese population. J. Affect. Disord..

[28] Hafeman D.M., Merranko J., Goldstein T.R., Axelson D., Goldstein B.I., Monk K., Hickey M.B., Sakolsky D., Diler R.S., Iyengar S. (2017). Assessment of a person-level risk calculator to predict new-onset bipolar spectrum disorder in youth at familial risk. JAMA Psychiatry.

[29] Moons K.G., Kengne A.P., Grobbee D.E., Royston P., Vergouwe Y., Altman U.G., Woodward M. (2012). Risk prediction models: II. External validation, model updating, and impact assessment. Heart.

[30] Moons K.G., Kengne A.P., Woodward M., Royston P., Vergouwe Y., Altman U.G., Grobbee D.E. (2012). Risk prediction models: I. Development, internal validation, and assessing the incremental value of a new (bio) marker. Heart.

[31] Salagre E., Dodd S., Aedo A., Rosa A., Amoretti S., Pinzon J., Reinares M., Berk M., Kapczinski F.P., Vieta E. (2018). Toward precision psychiatry in bipolar disorder: Staging 2.0. Front. Psychiatry.

[32] Fusar-poli P., Hijazi Z., Stahl D., Steyerberg E.W. (2018). The science of prognosis in psychiatry a review. JAMA Psychiatry.

[33] Fusar-Poli P., Hijazi Z., Stahl D., Steyerberg E.W. (2018). Early intervention in bipolar disorder. Am. J. Psychiatry.

[34] DelBello M. (2018). A risk calculator for bipolar disorder in youth: Improving the odds for personalized prevention and early intervention?. J. Am. Acad Child. Adolesc Psychiatry.

[35] Grande I., Magalhães P.V., Chendo I., Stertz L., Panizutti B., Colpo G.D., Rosa A.R., Gama C.S., Kapczinski F., Vieta E. (2014). Staging bipolar disorder: Clinical, biochemical, and functional correlates. Acta Psychiatr Scand..

[36] Rowland T., Perry B.I., Upthegrove R., Barnes N., Chatterjee J., Gallacher D., Marwaha S. (2018). Neurotrophins, cytokines, oxidative stress mediators and mood state in bipolar disorder: Systematic review and meta-analyses. Br. J. Psychiatry.

[37] Oliveira P.M.S., Oliveira P., Coroa M., Ribeiro J., Madeira N.G.G.F. (2018). Serum uric acid as a predictor of bipolarity in individuals with a major depressive episode. Bipolar Disord.

[38] Carvalho A.F., Firth J., Vieta E. (2020). Bipolar Disorder. N. Engl. J. Med..

[39] Kapczinski F., Magalhães P.V.S., Martinez V.B., Dias V.V., Frangou S., Gama C.S., Pinto A.G., Grande I., Ha K., Kauer-Sant’Anna M. (2014). Staging systems in bipolar disorder: An international society for bipolar disorders task force report. Acta Psychiatr. Scand..

